# Micronutrient Adequacy in Preschool Children Attending Family Child Care Homes

**DOI:** 10.3390/nu11092134

**Published:** 2019-09-06

**Authors:** Esther Cuadrado-Soto, Patricia Markham Risica, Kim M. Gans, Noereem Z. Mena, Carolyn Ellis, Carolina D. Araujo, Ingrid E. Lofgren, Kristen Cooksey Stowers, Alison Tovar

**Affiliations:** 1 Department of Nutrition and Food Science, Faculty of Pharmacy, Complutense University of Madrid, Plaza Ramón y Cajal S/N, 28040 Madrid, Spain; esther.cuadrado@ucm.es; 2 Center for Health Equity Research, Brown School of Public Health, Providence, RI 02912, USA; patricia_risica@brown.edu (P.M.R.);; 3 Department of Behavioral and Social Sciences, Brown School of Public Health, Providence, RI 02912, USA; 4 Department of Human Development and Family Studies, University of Connecticut, Storrs, CT 06269, USA; 5 Institute for Collaboration in Health, Interventions and Policy, University of Connecticut, Storrs, CT 06269, USA; kristen.cooksey@uconn.edu; 6 Department of Nutrition and Food Sciences, University of Rhode Island, Kingston, RI 02881, USA; mena23@my.uri.edu (N.Z.M.); carolyn_ellis@my.uri.edu (C.E.); caroldearaujo@my.uri.edu (C.D.A.); ingrid_lofgren@uri.edu (I.E.L.); 7 Department of Allied Health Sciences, University of Connecticut, Storrs, CT 06269, USA

**Keywords:** nutrient density, micronutrients, preschooler, family child care homes

## Abstract

Limited data is available on the micronutrient intake and adequacy in preschool children enrolled in family child care homes (FCCH). The goal of this paper is to describe the micronutrient adequacy relative to age-specific recommendations of preschool-aged children (aged 2–5 years) attending FCCH in Rhode Island (RI). Dietary data among younger preschoolers (aged 2–3 years), *n* = 245) and older preschoolers (aged 4–5 years), *n* = 121) in 118 RI FCCH (N = 366 children) were analyzed. Nutrient adequacy was assessed as the amount of nutrient per 1000 kcal of the diet that would meet the Institute of Medicine nutrient requirements (critical nutrient density), and it was compared to the observed nutrient densities of the children. The sodium:potassium ratio was also calculated. For most micronutrients, the observed density met or exceeded the recommendation, meaning the children’s intake was adequate. However, a high proportion of children had nutrient densities under the recommendation for vitamins D, E, K, and potassium (86.1%, 89.1%, 70.8%, and 99.2% of children, respectively). The mean vitamin B12, potassium, and zinc densities were statistically higher in younger vs. older preschoolers (*p* < 0.05 for all). Low densities in calcium and vitamins K and B5 were more frequent in older children vs. younger children (*p* < 0.05). In addition, older preschoolers had a higher sodium:potassium ratio than younger children (*p* < 0.05). The micronutrient intake density was adequate for most nutrients. However, intake of some nutrients was of concern. Further attention to training and compliance in FCCH may improve the diet quality of those cared for in these settings.

## 1. Introduction

Ensuring young children meet recommended nutrient intakes is important because early childhood is a critical time period for growth and development [1]. Although the United States (US) is a developed country, some children are not meeting the recommended micronutrient intakes for ideal growth and development [2,3]. Inadequacies in intakes for vitamin D, vitamin E, and potassium, as well as excessive intakes of vitamin A, zinc, and sodium, have been reported among US infants, toddlers, and preschoolers [3]. Micronutrient deficiencies can be detrimental to growth and future health [4,5]. For example, adequate calcium and vitamin D intake is essential to achieving appropriate bone mineralization [6,7], and intake of iodine, iron, zinc, and thiamine have been linked to cognitive development [8]. Additionally, deficiencies in vitamin D and to a lesser degree vitamin E, are associated with chronic diseases such as cardiovascular disease and diabetes [9,10,11,12]. Furthermore, inadequate potassium and excessive sodium intake among children can also contribute to the development of cardiovascular diseases [13,14]. Therefore, it is important to describe micronutrient intakes, especially among low-income and racial/ethnic minorities, whose diet does not meet national guidelines [15].

In the US, approximately 60% of preschool aged children receive external, non-parental care [16,17]. Of these, 24% are cared for by a non-relative in a family child care home (FCCH) [18]. Families who utilize FCCH are often low-income and more racially and ethnically diverse than those who attend center-based care [19]. Children who attend FCCH spend approximately 30 h a week in these settings, so they should be provided one-half or even two-thirds of their daily nutritional requirements [20]. While studies have reported that the diet of low-income, minority children is of poorer quality, with high consumption of energy dense foods and low consumption of nutrient dense foods [21,22], there is a lack of data on the micronutrient density in the diet of the preschoolers while in FCCH. Previous studies, through the analysis of foods and beverages served or the Healthy Eating Index (HEI) scores, have found that the diet quality of children who attend FCCH may be inadequate [23,24,25], but no studies have explored if children in this setting are meeting recommendations for micronutrients. In addition, no studies have explored the differences in micronutrient intake in this setting by child age. This is important because children’s specific needs evolve rapidly as they age and grow [8], and these changes in their requirements must be accompanied by a transition in their diet [26,27].

Therefore, this study aimed to describe the essential micronutrient intake of a diverse group of children attending 118 FCCH in Rhode Island and Massachusetts. We were also interested in exploring if the micronutrient intakes of preschoolers were adequate relative to their age requirements (2 to 3-year-olds vs. 4 to 5-year-olds).

## 2. Materials and Methods

### 2.1. Study Design

This study used baseline data from a cluster-randomized trial, ‘Healthy Start, Comienzos Sanos’, which is evaluating the efficacy of a multicomponent intervention to improve the food and physical activity environments of FCCH as well as the diet, physical activity, and screen time behaviors of the 2 to 5-years-old children in their care [15]. Details about the study recruitment, intervention, and evaluation are discussed elsewhere, [15] but the methods relevant to the current analyses are described below. Baseline data collection was conducted from January 2016 until July 2018. The Institutional Review Boards of Brown University, University of Rhode Island, and University of Connecticut approved all study procedures and materials. The participating family child care providers and parents of children provided signed consents. The study was registered in ClinicalTrials.gov with the identifier NCT02452645.

### 2.2. Setting

The study enrolled and measured 374 children attending 118 FCCH (approximately three children per FCCH) within 60 m of Providence, Rhode Island (RI).

### 2.3. Participants and Eligibility Requirements

To be eligible, the FCCH needed to be in operation for at least 6 months. The provider had to read and speak Spanish and/or English and care for at least one unrelated 2–5-years-old child for at least 10 h per week, who ate at least one meal and a snack per day at the FCCH. Eligible providers completed a 30 min baseline telephone survey followed by a 30 min in-person survey at the FCCH. Once parents of eligible 2–5-years-old children at that FCCH consented, a two-day observation was scheduled. Providers received $25 for completing the baseline survey and $50 for a two-day baseline observation.

### 2.4. Measures

#### 2.4.1. Demographics

Providers reported their gender, ethnicity, and race on the telephone survey and the following variables on the in-person survey—age, household income, marital status, education, years in the US, country of origin, years as a child care professional, number of children currently in their care (and how many are their own children or grandchildren), and whether the FCCH accepts Child and Adult Care Food Program (CACFP) benefits.

Providers also reported on the race and ethnicity of the children, information about the children’s age and gender, and the daily average number of hours that the children spent at the FCCH.

#### 2.4.2. FCCH Observation

Trained researchers visited the FCCH for two full days to observe the FCCH environment including the meals and snacks that the children consumed. On visit days, the research team arrived before the children had their first meal at the FCCH. Observers remained at the FCCH for as long as the children were there except during nap time. Each observer examined a maximum of three children from a convenient location so as not to interfere with their routine. If there were more than three children to observe, two observers attended the FCCH.

### 2.5. Foods and Beverages Served

The food and beverage consumption was calculated/estimated using the Dietary Observation in Child Care (DOCC) protocol developed by Ball et al. [28]. The DOCC protocol has demonstrated good validity and reliability for capturing the dietary intake of children in child care [28,29]. For this project, trained data collectors observed and recorded all meals and snacks served to the participating children across two full days of child care. Data collectors estimated the quantity of food and beverages served, added (i.e., second helpings), exchanged, wasted, and remaining following the end of each meal and snack to calculate the total quantity consumed by each child. Additional detail needed about recipes, preparation methods or brand names of food or beverages served were requested from the family child care provider.

The DOCC data, including foods consumed, the methods of food preparation, and the ingredients of the recipes, were entered into the Nutrition Data System for Research (NDSR) [30], which was used to determine the energy and nutrient content of all meals consumed throughout the day at the FCCH. Although most children were present for both day 1 and day 2 of observations, it was possible to have more or less children present on either of the days. As has been done in previous studies [31,32], if a child did not have two days of observation (*n* = 56, 15% of children), a single observed day was used for the analysis instead of averaging over two days.

#### 2.5.1. Nutrient Density and Adequacy of Meals

Given that this study captures children’s micronutrient intake only during their stay at the FCCH and not for 24-h, the micronutrient results are presented by 1000 kilocalories (kcals) (nutrient density) to be able to compare with recommendations [33,34]. To evaluate the nutrient adequacy of their diet, a critical nutrient density (CND) was calculated, a benchmark that has been used with infants and children [35,36,37,38]. The CND was defined as the amount of nutrient per 1000 kcal of the diet that would meet the Institute of Medicine (IOM) nutrient requirements [33]. To obtain nutrient densities, the nutrient intake provided at FCCH over the course of one day was used, divided by the total energy consumed in the same period, expressed per 1000 kcal. The nutrient density between the two days of data collection for each child was then averaged. This is consistent with the methodology used to calculate the Healthy Eating Index, where the amount of each nutrient consumed is expressed per 1000 kcal [39].

Based on the recommendations of the National Academy of Medicine (NAM)-previously called IOM- the following micronutrients of interest were selected—vitamins B1, B2, B5, B6, B12, niacin, folate, and vitamins A, C, D, E, and K [33]. The minerals calcium, copper, phosphorus, iron, magnesium, manganese, potassium, selenium, sodium, and zinc were also examined. These vitamins and minerals should be included on a daily basis in the diet to maintain children’s health [33].

The dietary reference intakes (DRIs) from the IOM were used as a comparison including the EAR (estimated average requirement) and AI (adequate intake). The EAR was used for vitamins A, B1, B2, B6, folate, vitamin B12, niacin, vitamin C, E, and D and the minerals calcium, phosphorus, magnesium, iron, zinc, copper, and selenium [33]. If a nutrient did not have an EAR, the AI was used as a reference for vitamin K, B5, sodium, potassium, and manganese [33].

Because the DRIs vary according to age, children were stratified into two age groups—young children aged 2 to 3 years and older children aged 4 to 5 years [33]. For energy requirements, the Daily Nutritional Goals for Age-Sex Groups provided by the 2015–2020 Dietary Guidelines for Americans [40] was used to calculate the CND. The recommended energy intake is 1000 kcal (children of 2 to 3 years), 1200 kcal (girls from 4 to 5 years), or 1400 to 1600 kcal (for boys from 4 to 5 years). The mean reference of 1500 kcal was used for boys of this age instead of the range.

The ratio of sodium:potassium was also calculated with a value greater than one signifying a less than optimal ratio as this ratio has been previously used to evaluate diet quality in children [13,41]. This ratio was used because it has a practical use for identifying need for sodium reduction and potassium increase [42]. The World Health Organization recommends a sodium:potassium ratio lower than 1. This recommendation refers to the sodium:potassium ratio expressed in mmol:mmol, its equivalence in milligrams is 0.6, therefore this cut-off point was used [43,44].

#### 2.5.2. Statistical Analyses

All analyses were conducted using SPSS 24.0. for Mac. The dietary data among two groups of children were analyzed—younger preschoolers (aged 2–3 years) and older preschoolers (aged 4–5 years) with age group as a categorical variable. The means and standard deviations were calculated for dietary outcomes. Nutrient density values, which were higher or lower than the recommendation, were determined by examining the mean and 95% CI as compared to the CND. If the CND for a given observed nutrient density was outside the 95% CI, the value was considered significantly different from the CND. For the children who did not meet the CND, the proportion of children under the recommended nutrient density for each micronutrient was calculated. To compare differences between the two age groups, a mean comparison *t*-test for continuous variables distributed normally was used, and Mann–Whitney U-test for variables distributed not normally. Chi-square test was used for categorical variables. A *p*-value < 0.05 was considered statistically significant.

## 3. Results

### 3.1. Descriptive Data

All 118 providers were female, 66.9% were Hispanic/Latino, most were born outside of the US (70.8%), approximately half reported a yearly income between $25,000 and $50,000 (47.5%), and had on average 12.9 ± 8.4 years of experience in child care (Table 1). Most of them participated in the CACFP (82.2%). On average, these providers had 7.7 ± 3.1 children in their care each day. Among the 366 preschoolers enrolled across 118 child care homes, 51.4% were female and the mean age of the children was 3.5 ± 1.0 years, 2.9 ± 0.6 years for the young preschoolers and 4.7 ± 0.5 for the older preschoolers. More than half of the children were considered Hispanic/Latino (57.1%) and they spent on average almost 8 hours per day (7.6 ± 0.9h) in the FCCH (Table 1).

Preschool children in this setting consumed 531.6 ± 268.5 kcal (children aged 2 to 3 years eating 487 ± 248.1 kcal and children aged 4 to 5 years eating 621.9 ± 286.3 kcal; *p* < 0.001), with 25.5 ± 9.8% of kcals from fat, 57.4 ± 12.2% from carbohydrates, and 17.2 ± 5.8% from protein. There was no difference in the number of meals or snacks consumed between the different age groups.

### 3.2. Nutrient Adequacy of the Diet

Table 2 and Table 3 show the mean nutrient density per 1000 kcals for vitamins and minerals. For almost all of the micronutrients, the observed density met or exceeded the recommendation for children aged 2–3 years and 4–5 years. The nutrients that had a lower mean observed density than recommended included vitamin D, vitamin E, and potassium (5.9 ± 3.5 µg/1000 kcal, 3.2 ± 2.5 mg/1000 kcal, 1638 ± 477 mg/1000 kcal respectively) in both age groups (Table 3). For these nutrients, a large proportion of children did not reach the recommended density (> 85%). On average the sodium:potassium ratio was higher than 1.0 in both age groups (mean ratio: 1.1 ± 0.6), which means that the intake of sodium is higher than the intake of potassium. There were no differences in the proportion of preschoolers with a ratio greater than one (*p* = 0.059), while the proportion of children with a sodium:potassium ratio above 0.6 and the mean sodium:potassium ratio were higher in the older children (90.9% vs. 81.6% and 1.12 ± 0.5 vs. 1.05 ± 0.6 respectively, *p* < 0.05).

### 3.3. Nutrient Data among Younger and Older Preschoolers

When comparing between the two age groups, the mean vitamin B12, potassium, and zinc densities were statistically higher in younger than older preschoolers (3.3 ± 1.6 µg/1000 kcal, 1670.2 ± 490.4 mg/1000 kcal and 6.2 ± 2.1 mg/1000 kcal vs. 3.0 ± 1.8 µg/1000 kcal, 1572.8 ± 443.6 mg/1000 kcal and 5.3 ± 1.5 mg/1000 kcal respectively, *p* < 0.05 for all). In addition, the proportion of children with densities of calcium and vitamins K and B5 below the recommendation was higher in older children as compared to younger children (27.3, 78.5 and 14% vs. 13.9, 66.9 and 6.9%, respectively *p* < 0.05). Other observed nutrient densities were similar between the two age groups.

## 4. Discussion

The goal of this paper was to describe the essential micronutrient intake of a diverse group of children attending FCCH in Rhode Island. We found that although most children had adequate intakes in almost all nutrients, they had lower intakes than recommended in vitamin D, E, K, and potassium. We also found differences in meeting recommendations by age groups, with older preschoolers less likely to be meeting the recommendations. Given that this is a vulnerable group of children at higher risk for micronutrient deficiencies [4], exploring inadequacies in micronutrient intake is of importance.

We found that on average, children attending FCCH consumed 531.6 ± 268.5 kcal (children aged 2 to 3 years eating 487 ± 248.1 kcal and children aged 4 to 5 years eating 621.9 ± 286.3 kcal; *p* < 0.001), demonstrating that children are eating approximately half of their daily recommended calories at child care. Our results are comparable to other studies that have found that preschool aged children consume approximately 500–600 kcal while in child care [24,45,46].

We also found that children had an adequate intake of most minerals and vitamins. However, the intake of vitamins D, K, E, and potassium was lower than recommended. In addition, the sodium:potassium ratio was > 1.0 for approximately half of the children and more than 80% of the preschoolers exceeded the WHO recommendation of 1 (mmol/mmol) expressed as milligrams (0.6 mg/mg) [43]. Our result that vitamin D intake was insufficient in most of the children is consistent with other studies which found that preschool children did not meet the recommendations for vitamin D [3,47,48]. In the Feeding Infants and Toddler study (FITS) [3], approximately 80% of children (under 48 months) did not meet vitamin D requirements, while Berner et al., [47] found that about half of preschoolers did not meet vitamin D requirements (including children taking and not taking supplements). The foods with the highest vitamin D content are fish, seafood, or foods fortified with vitamin D such as milk and milk drinks; thus it is possible that preschool children may not be consuming many of these foods while in child care. In a study conducted at 166 FCCH in North Carolina, with respect to the food served, FCCH providers nearly met HEI-2010 requirements for dairy products (9.6 ± 1.3 out of 10), however 30% of the dairy products served were not consumed [24]. In previous studies, using 24 h dietary recalls, almost half of the children aged 2 to 3 years in the US did not meet the recommended amounts of dairy foods (including foods given in regular child care outside the home) [49]. Given the important role that vitamin D plays in a child’s growth and development, it appears to be a nutrient of concern for young children.

About 70% and 90% of the children in this sample also did not meet the recommendations for vitamins K and E respectively. In the case of vitamin K, in contrast to our results, the FITS study found that a considerable proportion of children maintain an adequate intake of this vitamin (68–74%) [3]. The CND of vitamin K is based on an AI, not EAR, so it is not possible to evaluate the prevalence of inadequacy [3]. The proportion of a group that exceeds the AI reflects those who have adequate intakes. Our results could be explained by the fact that providers fall short in offering vegetables to children, as has been observed in other studies evaluating foods served at FCCH [23,24]. The main dietary sources of vitamin K are dark green vegetables [40] and Welker et al. found that 27% of children did not eat any vegetables throughout the day [50]. In particular, the number of children consuming green leafy vegetables was very low (15%), which could lead to a low intake of vitamin K. For vitamin E, previous studies have found fewer preschools with insufficient intakes of this mineral (approximately one-third) compared to our results [3]. A study carried out in Oklahoma child care centers (*n* = 25) found that both served and consumed amounts of vitamin E were inadequate [32]. The main sources of vitamin E are nuts and seeds and oils, [40] which may not be served in child care settings.

Like other vitamins described (vitamins D, E, and K), vitamin A is also a fat-soluble vitamin. In contrast to the other fat-soluble vitamins analyzed, the mean density observed was much higher than the recommended density. However, approximately 15% of preschoolers were found to have lower densities than recommended. Since vitamin A is a vitamin that can be stored in the body (as other fat-soluble vitamins) deficiencies in this vitamin would not be expected [51]. It has to be highlighted that food sources are the best route for good absorption and the safest way to obtain these micronutrients [51]. Also, other studies suggest that there are some gaps in the quality of the diet served in the FCCH. A 2012 study conducted in Washington FCCHs found that only 60.4% of the IOM recommendations were covered by the meals and snacks provided in the six micronutrients (vitamin E, iron, magnesium, potassium, zinc, and folate) evaluated after adjusting for 1000 kcals [45]. The diet quality in FCCH appears to be sub optional as observed in a North Carolina study, whereby child HEI while in child care averaged 58.9, far from the score of 80, which is recommended for a healthy diet [25].

Finally, children in our study had a higher than recommended sodium:potassium ratio (44.3% above one, 84.7% above 0.6) suggesting that the foods the children are consuming may be high in sodium and/or low in potassium. Our result is consistent with one study of 2882 preschoolers aged 1–3 years, and 1389 children aged 4–5 years, where the average sodium:potassium ratio was greater than one in both groups of age (sodium:potassium ratio 1.03 ± 0.01 in preschoolers aged 1–3 y and 1.30 ± 0.01 in preschoolers aged 4–5 years) [13]. Excess sodium intake is common among Americans of all ages [52,53]. The main sources of sodium in US preschools are cheese, bread, rolls, biscuits, processed and other meats, and salty snacks [13]. The sodium:potassium ratio is not just indicative of excess sodium intake but also inadequate potassium intake. A low potassium intake is associated with a low intake of vegetables (especially starchy vegetables), fruits and dairy products [40].

Some differences between the age groups are worth discussing. A greater proportion of young preschoolers (2–3 years) had inadequate vitamin D intakes as compared with older children (4–5 years). Although the requirement for vitamin D is the same in both age groups, since energy intake is higher in the older age group, it makes it more difficult for younger children to meet their requirements of vitamin D. However, in the case of vitamin K, B5, and calcium, a greater proportion of older children had inadequate intake as compared to younger children. This may be due to the fact that older preschool children have higher nutrient requirements (especially girls, who have a higher CND value than their peers and younger preschoolers except for vitamin D) and that providers may not be taking this into account when serving meals. A study conducted to analyze the meals that parents prepared for their children found that parents were not concerned about the increased needs of 4 to 5-years old compared to younger children [54]. This meant that older children had the same portions as younger children and that it was consequently more difficult for them to reach dietary recommendations [54].

In addition, in our study the sodium:potassium ratio was greater in the older age group. In a study about sodium and potassium intake in US children, the authors found that potassium intake was higher than sodium intake in American infants but that this reversed by age 4–5 years, in which the sodium:potassium ratio became greater than 1.0 [13]. In our study we also found this shift in the sodium:potassium ratio from younger to older aged children. Future studies might investigate the reason for this trend. Unlike centers, in FCCH, children of different ages are cared for together, combining children at various stages of growth and with different nutritional requirements. Future studies should explore if providers are aware of these different needs by age.

The results of this study may be informative for nutrition policy and education for providers. It appears that children’s overall intake of calories is adequate in child care, yet foods or beverages which are denser in nutrients of concern in this population (vitamin D, E and K, and potassium) need more focus in child care meals and snacks, as well as limiting foods with a high sodium density. To facilitate FCCH caregivers’ knowledge, examples of foods dense in these nutrients should be incorporated into care guidelines and trainings. Future research should confirm these results in larger populations and future studies should explore the impact of these deficiencies on child health outcomes.

There are many strengths to this study including the focus on data collection at FCCH, which have been understudied; the use of dietary intake data collected via observation, and the use of an up-to-date dataset of foods and nutrients through NDSR [30].

However, our study has some limitations. First, we did not calculate nutrient intake per day given that children in the FCCH had a different number of meals and snacks, hence the nutrient density calculation was more appropriate. Also, we did not collect dietary data on what was consumed at home and what possible nutrient supplements the children may have taken. We were however interested in the quality of the children’s diet at the FCCH, and not the diet quality at home. Future research should explore children’s dietary quality at both home and child care as well as the use of nutrient supplements in preschool children.

## 5. Conclusions

In our study, micronutrient density intakes were adequate for the majority of preschoolers enrolled. However, vitamin D, E, and K, potassium, and sodium are nutrients of concern that should be the target of further scrutiny and intervention to improve the diet quality of preschool children cared for in FCCH, especially in the transition from young to older preschool age. Recommendations and policies for improving nutrition in FCCH needed to meet nutrition standards and to fill gaps in the diets of preschoolers enrolled in these settings.

## Figures and Tables

**Table 1:** Descriptive data of Providers and Children.

Characteristic	Data, Mean ± SD or %
Providers (*n* = 118)	
Gender, % Female	100
Ethnicity, %	
Hispanic/Latina	66.9
Not Hispanic/Latina	33.1
Race, %	
White	43.2
Black or African American	15.3
Other/Unsure	41.5
Born outside of US, %	70.8
Years in US, (mean ± SD)	16.0 ± 13.7
Language spoken in child care, %	
English	26.3
Spanish	36.4
Both	28.8
Education level, %	
<High school diploma or GED	10.2
High school diploma or GED	32.2
Associates degree	39.0
Advanced degree	18.6
CDA (Child Development) Credential, %	
Yes	28.0
No	71.2
CACFP participation, %	82.2
Number of children in care, (mean ± SD)	7.7 ± 3.1
Years working in child care, (mean ± SD)	12.9 ± 8.4
Income, %	
Less than $25,000	13.3
$25,001–$50,000	47.5
$75,001 or more	15.8
Marital Status, %	
Single, never married	9.3
Married or living with partner	74.6
Divorced/Separated/Widowed	16.1
Children (*n* = 366)	
Age (years)	3.5 ± 1.0
2–3 years, %	66.9
4–5 years, %	33.1
Gender, %	
Male	48.6
Ethnicity, %	
Hispanic/Latino	57.1
Race, %	
White	54.1
Black or African American	15.3
Other/ Unsure	39.1
Hours spent in child care/day (mean ± SD)	7.6 ± 0.9

**Table 2:** Mean vitamin density per 1000 kcal and adequacy of vitamin intake by age group.

Vitamins	Critical Nutrient Density (Amount of Nutrient Recommended Per 1000 Kcal) ^a^			Density Observed Per 1000 Kcal, (Mean ± SD)			Children with Densities Below Recommendations, %		
	1–3 years	4–8 years, Boys	4–8 years, Girls	2–3 years (*n* = 245)	4–5 years (*n* = 121)	Total (*n* = 366)	2–3 years (*n* = 245)	4–5 years (*n* = 121)	Total (*n* = 366)
Vitamin A (µg RE/1000 kcal)	210.0	183.3	229.2	491.2 ± 256.8 †	473.2 ± 265.3 †	485.2 ± 259.4 †	15.7	13.8	15.1
Vitamin B1 (mg/1000 kcal)	0.4	0.33	0.42	0.9 ± 0.3 †	0.9 ± 0.3 †	0.9 ± 0.3 †	0.4	0.0	0.3
VitaminB12 (µg/1000 kcal)	0.7	0.67	0.83	3.3 ± 1.6 †	3 ± 1.8 † *	3.2 ± 1.7 †	4.5	7.4	5.5
Vitamin B2 (mg/1000 kcal)	0.4	0.33	0.42	1.5 ± 0.5 †	1.4 ± 0.6 †	1.4 ± 0.5 †	0.8	0.0	0.5
Niacin (mg/1000 kcal)	5.0	5.0	6.0	10.8 ± 4.1 †	10.9 ± 3.6 †	10.8 ± 4.0 †	4.1	0.0	2.7
Vitamin B5 (mg /1000 kcal)	2.0	2.0	2.5	3.4 ± 1.0	3.3 ± 1.0	3.4 ± 1.0	6.9	14.0 *	9.3
Vitamin B6 (mg/1000 kcal)	0.4	0.33	0.42	1.1 ± 0.4 †	1.0 ± 0.5 †	1.1 ± 0.4 †	2.4	1.7	2.2
Folate (µg/1000 kcal)	120.0	106.7	133.3	237.2 ± 96.2 †	221.1 ± 91.3 †	231.9 ± 94.8 †	4.5	5.8	4.9
Vitamin C (mg/1000 kcal)	13.0	14.7	18.3	68.4 ± 128.2 †	53.4 ± 52.5 †	63.4 ± 109.3 †	13.9	20.7	16.1
Vitamin D (µg/1000 kcal)	10.0	6.7	8.3	**6.0 ± 3.5 †**	**5.7 ± 3.4 †**	**5.9 ± 3.5 †**	90.6	76.9 ***	86.1
Vitamin E (mg α-TE /1000 kcal)	5.0	4.0	5.0	**3.3 ± 2.9 †**	**3.1 ± 1.3 †**	**3.2 ± 2.5 †**	91.0	85.1	89.1
Vitamin K (µg/1000 kcal)	30.0	36.7	45.9	32.0 ± 31.3 †	32.3 ± 26.7 †	32.1 ± 29.8 †	66.9	78.5 *	70.8

Asterisks indicate statistical significance of the difference between age groups: * *p* < 0.05, *** *p* < 0.001. α-TE: alpha-tocopherol. Nutrients in bold: the DRI is out of the 95% CI. **^a^** Amount of nutrient per 1000 kcal of the diet that would achieve the IOM nutrient requirements. We used the EAR or AI from the IOM, divided by 1000 kcal (children of 2 to 3 years), 1200 kcal (girls from 4 to 5 years), or 1500 kcal (for boys from 4 to 5 years). Observed Density was compared between age groups by *t*-test or Mann-Whitney U (†). Children with densities below recommendations were compared between age groups by chi-square test.

**Table 3:** Mean mineral density per 1000 kcal and adequacy of mineral intake by age group.

Minerals	Critical Nutrient Density, (Amount of Nutrient Recommended Per 1000 Kcal) ^a,b^			Density Observed Per 1000 Kcal, (Mean ± SD)			Children with Densities Below Recommendations, % ^c^		
	1–3 Years	4–8 years, Boys	4–8 years, Girls	2–3 years (*n* = 245)	4–5 years (*n* = 121)	Total (*n* = 366)	2–3 years (*n* = 245)	4–5 years (*n* = 121)	Total (*n* = 366)
Calcium (mg/1000 kcal)	500.0	533.3	666.7	841.6 ± 340	827.7 ± 357.1	837 ± 345.3	13.9	27.3 **	18.3
Copper (µg/1000 kcal)	260.0	226.7	283.3	559.1 ± 204.6 †	542.7 ± 156.9 †	553.7 ± 190.1 †	2.0	0.8	1.6
Phosphorus (mg/1000 kcal)	380.0	270.0	337.5	866.4 ± 241.4	831.6 ± 233.5	854.9 ± 239.1	2.0	0.8	1.6
Iron (mg/1000 kcal)	3.0	2.7	3.4	7.8 ± 3.7 †	7.5 ± 3.8 †	7.7 ± 3.7 †	1.2	0.8	1.1
Magnesium (mg/1000 kcal)	65.0	73.3	91.7	165± 41.4 †	160.1 ± 41.2 †	163.4 ± 41.3 †	0.4	0.0	0.3
Manganese (mg/1000 kcal)	1.2	1.0	1.3	2.2 ± 3.6 †	1.9 ± 1 †	2.1 ± 3 †	16.7	9.9	14.5
Potassium (mg/1000 kcal)	3000	2533	3167	**1670 ± 490**	1573 ± 444 *	**1638 ± 477**	99.2	99.2	99.2
Selenium (µg/1000 kcal)	17.0	15.3	19.2	58.9 ± 17.1†	59.5 ± 17.4 †	59.1 ± 17.2 †	1.2	0.0	0.8
Sodium (mg/1000 kcal)	1000	800	1000	1475.8 ± 479.2 †	1558.4 ± 491.3 †	1503.1 ± 484.1 †	11.8	6.6	10.1
Ratio Na/K (mg/mg)	<1	<1	<1	1.06 ± 0.59 †	1.12 ± 0.52 † *	1.08 ± 0.57 †	59.2	48.8	55.7
	<0.6	<0.6	<0.6				81.6	90.9 *	84.7
Zinc (mg/1000 kcal)	2.5	2.7	3.3	6.2 ± 2.1 †	5.8 ± 2.6 † *	6.1 ± 2.3 †	2.4	4.1	3.0

Asterisks indicate statistical significance of the difference between age groups: * *p* < 0.05, ** *p* < 0.01. Nutrients in bold: the DRI is out of the 95% CI. **^a^** Amount of nutrient per 1000 kcal of the diet that would achieve the IOM nutrient requirements. We used the EAR or AI from the IOM, divided by 1000 kcal (children of 2 to 3 years), 1200 kcal (girls from 4 to 5 years), or 1500 kcal (for boys from 4 to 5 years). **^b^** For the sodium:potassium ratio we use the cut-off point recommended by the World Health Organization [43,44]. **^c^** For the sodium:potassium ratio is the proportion of children that exceeds the cut-off point. Observed density was compared between age groups by t-test or Mann-Whitney U (†). Children with densities below recommendations were compared between age groups by chi-square test.
